# Emergency drills for agricultural drought response: a case study in Guatemala

**DOI:** 10.1111/disa.12316

**Published:** 2018-12-05

**Authors:** Anna Müller, Vesalio Mora, Edwin Rojas, Jorge Díaz, Obdulio Fuentes, Estuardo Giron, Ada Gaytan, Jacob van Etten

**Affiliations:** ^1^ Post‐Doctoral Fellow at Bioversity International, Costa Rica; ^2^ PhD student at Bioversity International and the Universidad de Costa Rica, Costa Rica, and Regional Planning Officer at the Ministry of Agriculture and Livestock, Costa Rica; ^3^ Vice‐Minister for Sustainable Development at the Ministry of Energy and Mines, Guatemala; ^4^ Program Officer at the Rural Extension Unit at the Ministry of Agriculture, Livestock and Food, Guatemala; ^5^ Assistant‐Director of the Unit for Integrated Disaster Risk Management at the National Coordination for Disaster Reduction, Guatemala; ^6^ Project Coordinator at the Tropical Agricultural Research and Higher Education Centre, Costa Rica; ^7^ Project Coordinator for Disaster Risk Reduction at Acción contra el Hambre, Guatemala; ^8^ Senior Scientist at Bioversity International, Costa Rica.

**Keywords:** climate adaptation, cyclical drought, emergency drill, institutional capacity, slow‐onset disasters

## Abstract

Drills are an important element of disaster management, helping to increase preparedness and reduce the risk of real‐time failure. Yet, they are not applied systematically to slow‐onset disasters such as a drought, which causes damage that is not instantly apparent and thus does not solicit immediate action. This case study evaluates how drills inform institutional responses to slow‐onset disasters. It spotlights Guatemala, a country where drought has severe impacts on livelihoods and the food security of small farmers. By implementing part of the Ministry of Agriculture, Livestock and Food's institutional response plan for drought, it explores how drills can help to detect issues in emergency response and to foster an institutional focus on improvements in preparedness. The results reveal that drills alone do not trigger institutional improvements if unsupported by a wider strategy that seeks to enhance capacities and protocols. These findings are valuable, however, in making problems transparent and in creating the space for discussion.

## Introduction

Drought is accorded much lower priority in disaster management than other types of disaster (Wilhite, 2005). The particular nature of drought‐induced emergencies makes them difficult to manage. In comparison to abrupt emergencies such as earthquakes, floods, typhoons, or volcanic eruptions, drought has a slow onset and is less visible to the public eye. It can be reasonably well predicted in many areas of the world, but it is difficult to determine objectively in real time the precise moment at which a dry period becomes an emergency. When a drought starts, its effects on human well‐being and the environment are not immediately clear.

Drought can cause substantial damage over large areas, but does little damage to easily observable assets, such as homesteads or public infrastructure. Instead, drought‐related damage comprises largely losses to harvests and livestock that are difficult to quantify without detailed studies. Knock‐on effects on food availability tend to become acute only some months after the actual event begins. The ambiguous and slow nature of drought often precludes a brisk and decisive emergency response and hinders the development of proactive drought management (Wilhite, Sivakumar, and Pulwarty, 2014). To make matters worse, frequently when the extent of the drought has become apparent and decision‐makers start to panic, it begins to rain. The emergency swiftly loses its political urgency and decision‐makers retreat into apathy. This vicious circle hinders the development of a resilient farming sector and augments vulnerability to future droughts (Wilhite, Sivakumar, and Pulwarty, 2014).

Droughts become a disaster to society only when there are no mechanisms in place for response, coping, and mitigation. Early intervention is decisive to avoid an emergency, which can be managed with available resources, becoming a disaster, where the ramifications may be beyond the capacities of the institutions operating in the affected area. In many susceptible regions, projections foresee an increase in droughts (Wilhite, Sivakumar, and Pulwarty, 2014). This underlines the importance of devoting greater attention to drought management.

The shift, from reactive crisis response to proactive risk management, requires several changes, including the development of institutional capacity and the provision of timely information to key decision‐makers (WMO and GWP, 2014). It is crucial to create learning opportunities for organisations working in drought response and a sound situation assessment to target interventions to reduce the impacts of drought (Hedlund, 2007).

An important approach to evaluate and improve the organisational capacity of a public institution to respond to an emergency is the use of a *drill* or a *simulation* (Lee et al., 2009).^1^ Drills are a compulsory part of many emergency response strategies. They can be used to evaluate the preparedness of a hospital to tackle a catastrophic accident with many casualties or in schools to train staff and students in the event of an earthquake or a fire (Lee et al., 2009). Drills have not been used extensively, though, in drought management.

The aim of this study is to evaluate how drills can inform institutional responses to slow‐onset disasters such as a drought. To this end, the research team organised two emergency drills in Guatemala, each implementing a different step of the existing institutional drought response protocol. This case study explores how drills can help to detect issues in drought emergency response and to yield an institutional focus on possible improvements in preparedness.

## Background

### Building institutional capacity in drought management using drills and simulations

Climate projections foresee a rise in the frequency and severity of droughts in many regions of the world (Wilhite, Sivakumar, and Pulwarty, 2014), underscoring the importance of devoting greater attention to building institutional capacity for proactive drought management. Drills offer a way to do this because both individual and institutional disaster management learning benefit from games and simulations (Crookall, 2010; Hofstede, de Caluwe, and Peters, 2010).

With drills, an organisation can avoid trial‐and‐error learning during an actual emergency, which usually comes at a high economic cost and can lead to irreversible damage or even the loss of human life (Nathan and Kovoor‐Misra, 2002). Drills serve to detect the potential failings of communication and coordination and provide opportunities for the improvement of emergency response protocols. They produce a sense of ownership and shared focus, unlike a paper protocol. One of the strengths of drills and simulations is that they allow one to evaluate the capacity and knowledge of individuals and organisations, as well as their ability to act collaboratively (Hofstede, de Caluwe, and Peters, 2010).

One endemic issue in emergency response is the learning–action gap: knowing about what is good and effective in emergency response does not necessarily result in behavioural change among individuals or organisations. This lacuna is a product of the incentive environment that shapes individual and organisational behaviour (Pelling, 2007).

Simulations seek to close the learning–action gap, linking tacit and formal knowledge through action and experience (Duke, 2004). That said, simulation exercises need to adhere to a certain structure to ensure that the experience translates into a proper learning process (Crookall, 2010; Hofstede, de Caluwe, and Peters, 2010). Simulations or drills are not only about playing in a safe setting: they start with a careful design process, including participatory elements, which sets the basis for the event. The drill itself includes participants and observers and ends with a thorough debriefing process to ensure that the experience translates into a learning process (Crookall, 2010; Hofstede, de Caluwe, and Peters, 2010). Debriefing and reflection are at the core of the experiential learning process (Kim, 2014).

Drills and simulations as a tool to inform institutional emergency response have been studied in different fields of disaster risk management. If properly designed, multi‐agency exercises or simulations can create mechanisms for learning (Andersson, 2016). This includes going beyond the focus on action and requires participants to invest valuable time in debriefing and discussion after the event. In this way, drills create a space for making explicit prior knowledge and for critical reflection.

A study using drills to test intra‐organisational emergency preparedness in the transportation sector found that the role of the external observer is particularly important (Yoon et al., 2008). The studied drills were an effective instrument for enhancing performance during emergencies. They permitted the evaluation of institutional capabilities at low cost and facilitated observation of individual and institutional emergency responses and consideration of the strengths and weaknesses of the organisation.

Bharosa, Lee, and Janssen (2010) used observations during an emergency drill and survey data subsequently to understand how actors with different institutions share and exchange information during a simulated emergency. Observations throughout the drill allowed them to obtain detailed insights into the complexities of multi‐agency emergency response, which would not have been possible using only survey data gathered afterwards. Analysing the use of simulations in the disaster response of the United Kingdom, Kim (2014) suggests that such exercises can inform the adaptation of manuals, plans, and skills, assuring the degree of institutional flexibility necessary for targeted disaster response. As Bharosa, Lee, and Janssen (2010) note, the strength of emergency drills is that they afford direct observation of real‐time behaviour.

Drills for drought management, however, are virtually non‐existent. For drought management in the context of the United States, Mason and Verner (2008) describe ‘table‐top’ exercises, in which participants respond to drought in a simulation that takes them through an accelerated unrolling of events. These exercises were found to be especially helpful in investigating and addressing inter‐institutional communication, coordination, and planning. Hill et al. (2014) present different crisis management tools via simulation games to observe decision‐makers’ behaviour in a simulated emergency. But these exercises are not drills.

Even though drought is a slow‐onset event and institutional responses are often indecisive, when individual responses are needed, they tend to be executed under great time pressure. Once it passes a tipping point, a drought represents a serious threat to agriculture, food security, and human life and warrants immediate attention. When a state of alert or emergency has been officially declared, the priorities are to provide credible data on the extent of damage and loss, and to instigate a swift response. Real‐time drills could make a distinctive contribution to drought management that has not been properly evaluated to date.

### Drought in Guatemala

Guatemala is a drought‐prone country (Wilhite, 2005). It is characterised by a high incidence of poverty and food insecurity, with a social system that is highly unequal in terms of economic opportunities and weak public institutions. The country has to deal with multiple environmental and social conflicts (Pillay, 2006; The World Bank, 2009),^2^ and its agricultural sector comprises a large proportion of small‐scale subsistence agriculture and livestock production with low levels of technological sophistication (FAO, 2014).

Guatemala's climate is divided into a dry (November–April) and a rainy (May– October) season with some variation in timing between regions and years. In the area of the present study, Chiquimula, average rainfall ranges from 600–1,200 millimetres per year in different municipalities. The distribution of rainfall is bimodal: two peaks of rainfall manifest, separated by a dry spell called *canícula* that normally transpires between June and August (GWP, 2014). Drought, in this climate, refers mainly to the variation in the duration of this dry spell. The length of the *canícula* does not necessarily affect average annual precipitation and the water recharging of the region. However, this dry spell occurs when the main food crops are still growing and it becomes a hazard to agriculture and food security if its length extends beyond what is expected based on historical experience and patterns.

Variations in the start, duration, and end of this dry spell challenge small farmers’ planning decisions about when to plant their basic staple food crops, maize and beans (GWP, 2014). The prolonged dry period is cyclical in nature and is highly related to the El Niño–Southern Oscillation (ENSO) (ACF, FAO, and ECHO, 2012). Cyclical drought thus poses a serious threat to the livelihood strategies and food security of farmers, since they lack strong coping mechanisms as a result of poverty and a dearth of specific knowledge (ACF, FAO, and ECHO, 2012).

An estimated 1.3 million people in Guatemala were affected by an unusual drought in 2015, with some 720,000 in immediate need of food assistance (UN‐OCHA, 2015). Nevertheless, the Ministerio de Agricultura, Ganadería y Alimentación de la República de Guatemala (MAGA)^3^ did not issue any official information on damage to and losses in basic grain production and the Government of Guatemala did not declare an official emergency (ACF, 2015b). Key informants from the non‐governmental sector claimed that the institutional response of governmental bodies was insufficient, uncoordinated, and inefficient. Then, with the first rains, the situation in Guatemala eased and public actors diverted their attention to other urgent matters. This behaviour is known as the ‘hydro‐illogical cycle': as soon as it starts raining, the emergency loses its potential urgency and decision‐makers fall back into apathy (Wilhite, 2012).

Forecasts suggest that climate change will intensify droughts induced by the El Niño phase of the ENSO cycle in the next few decades (Imbach et al., 2010). Guatemala is the country with the largest area affected by cyclical droughts in Central America (ACF, FAO, and ECHO, 2012) and was among global water and security hotspots in 2016, as the effects of the 2015 drought, government failure, violence, and a weak economy threatened to generate social tension and increase migration (LaFond and Kozacek, 2016).

Such events pose a serious risk to small farmers’ agricultural production, their livelihoods, and the food security of a great proportion of rural families in the region (Imbach et al., 2017), and indicate the urgent need for the country to develop a sound climate risk management strategy. For instance, early interventions (such as changes in the sowing period) or early responses (such as targeted food aid) can mitigate the impacts of drought.

### Institutional context for drought response in Guatemala

Effective drought management policies aim to decrease vulnerability to and the consequences of drought, and they are in place and operational before the full severity of an event is known. In Guatemala, public institutions together with non‐governmental aid agencies are increasingly working towards a more integrated drought risk management approach (CONRED, 2015).

To address the risk presented by an agricultural drought to the production system and rural food security, MAGA developed a Plan Institucional de Respuesta (PIR) in 2012. The objective of the institutional response plan is to reduce the impacts of drought on agricultural infrastructure, crops, and human life (MAGA, 2012). In case of an abnormally long period without rain, the PIR defines a series of actions and protocols to be followed to evaluate the situation in the agricultural sector and to inform decision‐makers. The institutional steps establish communication flows and the chain of command between the different decision‐making levels in MAGA.

Authorities decide whether or not to declare an emergency based on the information produced through the process. Declaring an emergency permits the release of national and international emergency funds to respond to the drought to ensure food security. The successful implementation of PIR protocols is critical for a proactive drought management strategy, as the information gathered in the field is decisive in order to act and react in time.

The PIR is embedded in national legislation on disaster prevention and reduction, which spans several ministries and national institutions, and responds to the Plan Nacional de Respuesta (PNR). The Coordinadora Nacional para la Reducción de Desastres (CONRED), the national coordination entity for disaster reduction, is responsible for the implementation of the PNR, the national response plan, and coordinates public and private institutions, civil society, and international donors with respect to disaster response and prevention in Guatemala (CONRED, 2011). CONRED has started to work on plans for drought response and damage prevention (CONRED, 2015), an important step towards incorporating a slow‐onset disaster in an integrated risk management framework.

**Figure 1:**
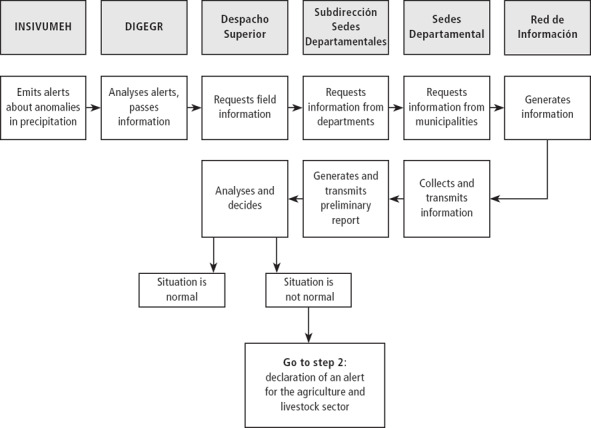
Step 1 of the PIR: monitoring the situation in the field **Notes:** INSIVUMEH=Instituto Nacional de Sismología, Vulcanología, Meteorología e Hidrología (National Institute for Seismology, Vulcanology, Meteorology and Hydrology); DIGEGR=Dirección de Información Geográfica, Estratégica y Gestión de Riesgo (Directorate of Geographic, Strategic and Risk Management); Despacho Superior=ministerial central decision‐making unit; Subdirección de Sedes Departamentales=sub‐directorate of the department dependencies; Sedes Departamentales=department dependencies; and Red de Información=key informants network. **Source:** authors, simplified overview, adapted from MAGA (2012).

The response protocol in case of droughts is composed of five steps: (i) monitoring of the situation in the field; (ii) declaration of an alert; (iii) declaration of an emergency; (iv) actions during the drought; and (v) actions after the drought. All five steps follow the same chain of command as presented in Figure 1. After every step, the central decision‐making unit (minister and vice‐ministers) decides whether or not to continue with the next step in the chain, based on the information gathered at the local level.

This study evaluated the first two steps of the PIR's drought protocol. The first step consists of preliminary monitoring of losses and damage in the agriculture and livestock sector of the affected region after a prolonged period of no rain. This information is used by the central decision‐making unit, the Despacho Superior (Office of the Minister), to decide whether or not the situation is normal. In the case of abnormal circumstances, the second step of the response protocol comes into effect: the declaration of an alert owing to severe drought conditions.

Step one commences when the Instituto Nacional de Sismología, Vulcanología, Meteorología e Hidrología (INSIVUMEH)^4^ provides information on abnormally low precipitation. The Dirección de Información Geográfica, Estratégica y Gestión de Riesgo (DIGEGR)^5^ passes this alert to the Despacho Superior. Based on this information, the latter decides whether or not to request more detailed information from the department coordination unit, the Subdirección de Sedes Departamentales.^6^ This unit passes the information request to the head of the department units in the affected region. He/she then dispatches extension agents to evaluate the situation in the field using an established network of farmers. A report based on the field evaluation goes back following the same chain of command until it reaches the Despacho Superior (see Figure 1; see also MAGA, 2012), which subsequently decides whether or not the situation is normal. If MAGA declares it to be abnormal, step two of the protocol applies.

**Figure 2:**
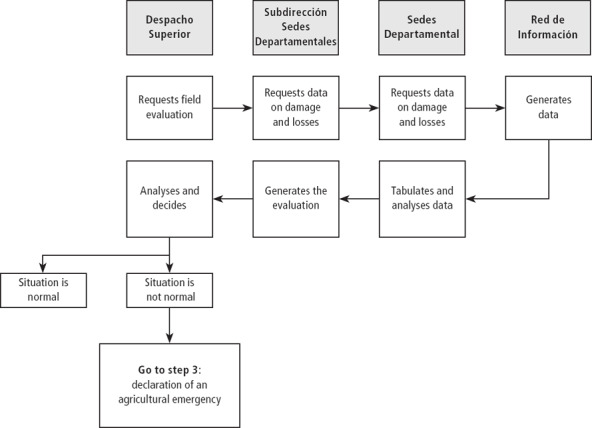
Step 2 of the PIR: declaration of an agricultural emergency **Notes:** Despacho superior=ministerial central decision‐making unit; Subdirección de Sedes Departamentales=sub‐directorate of department dependencies; and Red de Información=key informants network. **Source:** authors, simplified overview, adapted from MAGA (2012).

Step two begins with the Despacho Superior requesting more detailed information on damage and losses in the affected regions. This request is passed through department coordination and department dependencies to the informant networks. Extension agents gather detailed information in the field, which goes back to the Despacho Superior through the same nodes (see Figure 2). At the end of step two, a decision is made on whether or not to declare an emergency in the agriculture and livestock sector. If the decision is ‘yes', step three of the protocol starts.

As outlined above, the slow‐onset character of a drought makes it difficult for decision‐makers to decide on the status of an emergency and interventions. The PIR represents the institutional guide to cope with the challenges that drought poses.

### Study area

**Figure 3:**
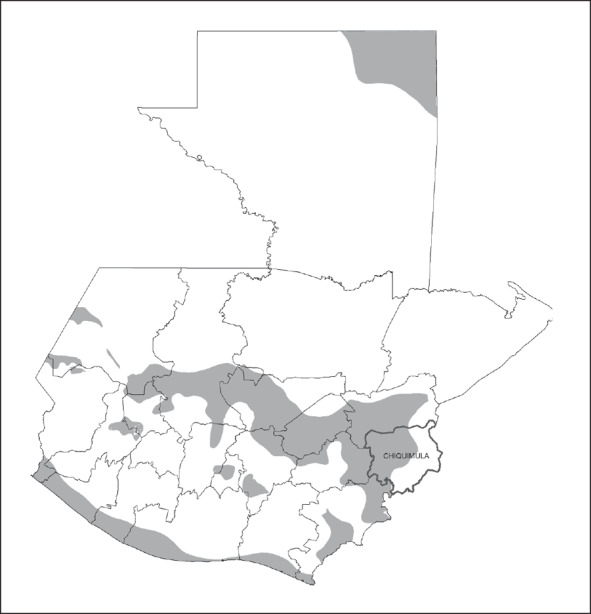
Drought‐prone zones and study region in Guatemala **Source:** first author (Anna Müller), based on information acquired from the Instituto de Agricultura, Recursos Naturales y Ambiente (IARNA), 2009.

The study region forms part of the Central American dry corridor. The emergency drills were conducted in collaboration with the MAGA department dependency of Chiquimula (see Figure 3). Chiquimula and the Guatemalan dry corridor suffered four unusually extreme drought periods between 2009 and 2015 (ACF, 2015a).

The study region is characterised by small‐scale subsistence‐based staple food production, a high rural poverty rate, and major vulnerability to food insecurity among the rural population (FEWS NET et al., 2016). The poorest members of the rural population are not able to sustain their families throughout the year and depend on income as agricultural day labourers. Temporary migration to other parts of Guatemala, as well as to El Salvador and Honduras, has been an integral part of the livelihood strategy of farm households in Chiquimula. In recent years, however, a severe coffee rust epidemic in the region has reduced opportunities for seasonal work considerably, with adverse effects on food security and increased vulnerability to drought (ACF, 2015a; Avelino et al., 2015).

## Methodology

### Drill design

Two drought emergency drills were executed in various municipalities in the department of Chiquimula: one in 2014, and the other in 2015. They were prepared at meetings involving experts from CONRED and MAGA, as well as from Action Against Hunger (ACF), the Centro Agronómico Tropical de Investigación y Enseñanza (CATIE), and Bioversity International.^7^

The first emergency drill was a simulation of step one of the PIR (that is, monitoring of precipitation and the situation on the ground). The second emergency drill was a simulation of step 2 of the PIR (that is, the declaration of an alert owing to an agricultural drought). In both instances, a field evaluation took place, organised in the same way. The emergency drill was partial, focusing on communication between the department dependency and the extension agents and the field evaluation of damage. The component involving ministerial decisions was omitted.

Two groups of people participated in the drill. The first group was composed of MAGA technical staff at all levels of command and from various positions along the information chain, including staff from the headquarters of the ministry and the regional dependency, as well as agricultural extension agents employed by MAGA. The second group was composed of observers and evaluators who accompanied the extension agents during the field evaluation in pairs; representatives of the other participating organisations assumed this role.

Both drills lasted 1.5 days. One day was spent on execution of the emergency drill in the field and half a day was spent on evaluation of the drill and its results with participants and key stakeholders.

Preparations for the drill involved creating a simulated damaged plot for the extension agents to assess. A number of farmers were invited to function as informants during the endeavour. Farmers were purposely selected based on the characteristics of their farms: relatively good access and cultivation of beans or maize, the main staple crops in the area. During the first drill, farmers were beneficiaries of projects led by ACF; during the second drill, they were members of MAGA's extension network. In 2014, the drill covered three municipalities, with three farmers serving as key informants in each municipality, whereas in 2015, the drill spanned six municipalities, with two‐to‐three key informants in each municipality. To simulate crop damage on the farms, every external group evaluator received a poster with pictures of two different degrees of damage to plants and a reference picture of non‐damaged plants. Different coloured tape represented different types of damage: black indicated damage of grain, whereas red designated damage to the development of the plant. The coloured tapes were randomly placed on a certain number of plants to simulate different degrees of damage to crops in the fields visited by the extension agents.

A preparatory meeting for all technical personnel involved at the local level was held before the actual drill. All persons engaged in the initiative at different institutional levels were briefed beforehand. The extension agents were told to act within their actual professional role. In evaluating production damage and losses, the extension agents interviewed the selected informants, visited plots with simulated damage, and talked to community leaders. Official MAGA procedures and forms were used to gather the data. Every participant received a folder with instructions on and a timetable for the drill.

### Research approach

A combination of data gathered through participatory observation during the drill and self‐assessment by the participants was used to assess the results of the drill. The mixture of techniques made the exercise more transparent. Moreover, employing only self‐assessment would have been less reliable (Kobes et al., 2010). Participatory observation allows one to understand how people behave during an event and is a practical means of collecting data throughout an operation (Bharosa, Lee, and Janssen, 2010). Groups of observers and extension agents indicated the advantages and disadvantages of the drill and the protocol, as well as the strengths and weaknesses of the procured results. Participants, drawing on their field experience, also elaborated recommendations for MAGA decision‐makers.

Detailed notes were made regarding observations of extension agents’ behaviour and actions during the drill. Reflections on other groups were shared by observers from the other participating organisations, which were recorded during the evaluation meeting at the end of the exercise.

**Table 1:** Communication flow during the drill (based on the first exercise)

DAY 1	
09:05	Drill starts
09:35	**From MAGA central level to MAGA department dependency, Chiquimula**
	Request for a report on the impact of the drought on agricultural crops in the area via e‐mail.
	Instructions to organise the working groups and to pay attention to further instructions that will be sent by e‐mail.
	The message starts and ends with the declaration: This is a drill!
10:05	**From MAGA central level to MAGA department dependency, Chiquimula**
	Instructions concerning the three communities selected for evaluation throughout the drill are sent by e‐mail.
	The message starts and ends with the declaration: This is a drill!
10:15	Extension agents and external observers depart for the field evaluation.
11:00	**From MAGA central level to MAGA department dependency, Chiquimula**
	E‐mail with details on the community leaders that extension agents should contact to collect information on damage and losses owing to the drought.
	The message starts and ends with the declaration: This is a drill!
11:25	**From extension agents to MAGA department dependency, Chiquimula**
	One group of extension agents states that it was unable to contact the leader of the community who was supposed to be visited. Cellular telephone coverage in the corresponding community is very weak.
13:55	**Department dependency, Chiquimula, to extension agents**
	One group of extension agents has not yet reached the corresponding community. A department‐level representative tries unsuccessfully to make telephone contact with the group.
14:20	**Department dependency, Chiquimula, to extension agents**
	A department‐level representative is managing to communicate with the group. The group informs him/her of its assumption that the members should visit a community of their choice. The group states that it did not receive the e‐mail with the instructions on which communities to visit throughout the drill. The group is advised to go to the initially selected community.
14:30	**Department dependency, Chiquimula, to extension agents**
	Extension agents must send a preliminary report by 17.30.
	The e‐mail starts and ends with the declaration: This is a drill!
16:10	**Department dependency, Chiquimula, to extension agents**
	Extension agents receive the e‐mail confirming 17.30 as the deadline for submission of the preliminary report.
	The message starts and ends with the declaration: This is a drill!
17.30	**Department dependency, Chiquimula, to extension agents**
	E‐mail sent to the extension agents to confirm the submission of all reports. The extension agents will receive further instructions on how to proceed with the evaluation at 08:00 on the following day.
	The message starts and ends with the declaration: This is a drill!
**DAY 2**
08:00	**Department dependency, Chiquimula, to MAGA central level**
	E‐mail sent to confirm that the field phase of the information collection process has finished. The extension agents have to submit their information for the final report by 12:00.
	The message starts and ends with the declaration: This is a drill!
08:45	**MAGA central level to department dependency, Chiquimula**
	E‐mail sent to confirm receipt of the final report. With the information received, the drill is officially over.
	The message starts and ends with the declaration: This is a drill!

a **Source:** authors’ elaboration based on field protocols.

Observational and self‐assessment data were appraised using a qualitative analysis approach (Lecompte, 2000). The data were cleaned in preparation for analysis, and were structured and ordered to pinpoint groups of items in the observations and self‐assessments. In this way it was possible to group data and observations for further interpretation.

### Drill execution

The same methodology and communication flows identified by the PIR were followed during both drills, which do not differ significantly between steps one and two (see Figures 1 and 2). Table 1 gives the reader an idea about the course of the first drill to exemplify the event.

The drills started with a request from the coordination unit of the central offices of MAGA to provide information on damage to and losses in basic grain production owing to an extended drought. All instructions from the higher hierarchical level were clear about the nature of the exercise. In addition, the department coordination of MAGA was in constant contact with extension agents in the field via e‐mail and cellular telephone. Both drills finished with the successful submission of a situation report on damage to and losses in grain production by MAGA extension agents to their superior.

The first drill involved 20 participants: six extension agents, who operated in pairs; nine external observers; and five people from the organising committee. Three external observers remained in the coordination unit and six observers accompanied each of the three groups of extension agents. The second drill involved 27 extension agents and 14 external observers. Two observers remained in MAGA's dependency to observe coordination of the exercise. The other observers accompanied the six groups of extension agents to the field.

## Results

### Participatory evaluation of the drills

In a participatory group exercise, extension agents and external observers and evaluators discussed the strengths and weaknesses of implementation of the PIR in the drill and suggested recommendations. The results of both drills were analysed together, as the strengths, weaknesses, and recommendations identified by the participants can be subsumed under the same topics.

#### Strengths identified by the participants

- **Extension system**: the extension system with motivated field staff who work with an extensive network of farmers in a relationship of mutual trust was considered to be an important prerequisite for successful implementation of the protocols. The participants felt that the relationship of trust with farmers makes the information more reliable, as they are more willing to participate.
- **PIR instructions**: the participants stated that the PIR clearly defines the chain of command and information flows between the different hierarchical levels of MAGA, aiding implementation of the protocols.
- **Inter‐institutional coordination**: well‐functioning coordination among institutions at the local level was identified as essential for successful implementation of the protocols.

#### Weaknesses identified by the participants

- **Lack of knowledge and experience of the PIR**: one reason that the participants identified for a lack of knowledge of the PIR and experience of its implementation was the high turnover of MAGA technical staff. Communication channels within the MAGA structure are weak and decisions made at the central level do not necessarily reach the dependencies.
- **Unrepresentative data**: although there is an existing survey instrument for data collection for damage and loss evaluation, the extension agents said that they were not trained in its application. They were using individual methods in the field. Extension agents clean and manipulate the data gathered in the field before they send them to the central agency. The participants felt that this could influence the decisions made at the central level (politicisation of the data). The number of communities, farmers, and plots visited during the drill was very small and the participants questioned the representativeness of the damage evaluation during the endeavour.
- **Poor access to supporting infrastructure**: the participants believed that they have poor access to supporting infrastructure, impeding good implementation of the protocols. They noted that they lack access to official meteorological data and supporting information, such as georeferenced data and maps. MAGA does not have enough vehicles to mobilise extension agents in the event of an emergency and many MAGA dependencies do not have well‐functioning information technology infrastructure.
- **High staff rotation and institutional ties based on personal relationships**: although good inter‐institutional coordination in Chiquimula was mentioned as a strength, the participants claimed that contacts between institutions and organisations depend mainly on the personal contacts of the technical staff. Inter‐sectoral communication in an emergency is not institutionalised and not mentioned in the PIR. Changes in staff thus directly affect the coordination of local institutions in an emergency. Paternalistic structures and frequent changes in personnel and of programmes at all levels of the public administration were seen as threats to MAGA's effort to improve the response to extreme droughts and institutional capacity‐building.
- **Raising false expectations among farmers**: the participants were worried that implementation of the protocols was leading to high expectations among the farmers who were visited during the drill. Visiting farmers to evaluate damage on their farms raises expectations about help, especially as the drill was implemented during a period of drought. However, the decision on measures to mitigate the impacts of drought was not part of the exercise. In a real emergency, the extension agents do not have the power to decide on mitigation measures, placing them in a difficult position vis‐à‐vis the farmers they are visiting.^8^
- **Poor institutional preparation**: the extension agents raised concerns about feeling generally unprepared regarding the threats posed by agro‐climatic events, and they expressed doubts about institutional preparedness.
- **Missing communication flow**: the extension agents complained that information on the results of the damage evaluation does not flow back from the central level to them. It seemed to them that they are sending the information into a black hole. This is demotivating, as the extension agents never receive feedback on whether or not their efforts to collect information have been useful and the information has been used by decision‐makers. The information gathered also has direct value for their work in the field.
- **Infrastructure**: the extension agents complained that road infrastructure is poor, making it difficult to reach remote villages. Cellular telephone coverage is also poor in remote villages.

#### Recommendations

Based on the strength–weakness analysis, the participants identified several strategic activities to enhance the implementation of the PIR:

- **Initiate institutional capacity‐building**: the participants saw opportunities to improve the decision‐making and information management capacity of MAGA through the drill by implementing these exercises regularly, as well as in other regions, thereby initiating a process of institutional learning. They noted that MAGA should provide capacity‐building to enhance the knowledge of its technical staff of the PIR and the implementation protocol.
- **Define the institutional evaluation method**: the homogenisation of the instruments used to evaluate damage and loss in the field (indicators and quantitative procedures for estimating plant and yield loss, for instance) would lead to more consistent and comparable estimates of the severity of a drought.
- **Create supporting infrastructure**: mapping the most drought‐vulnerable zones in Chiquimula would make logistics easier during an emergency, and could help in acquiring a representative sample. The participants underscored the need for more meteorological weather stations in the area to improve MAGA's access to additional and reliable information on local agro‐climatic conditions.
- **Provide capacity‐building to farmers**: the participants said that improving farmers’ knowledge of agro‐climatic features could enhance the information that they provide to the extension agents.
- **Improve inter‐institutional cooperation**: collaboration between institutions depends greatly on personal contacts. The participants saw an opportunity, therefore, to improve implementation of the PIR by officially incorporating other local institutions in MAGA's disaster response plan. They developed the idea of a local drought entity that could coordinate this effort.

### Participatory observations of both drills

The participants in the second drill were not informed about the results and recommendations of the first drill. In both instances, the staff members responsible for coordination within MAGA did not dedicate much time to organising and coordinating before and during the drill. There appeared to be a general lack of institutional commitment, affecting the course of the event. Contrary to the extension agents, instructions were missing on how to conduct the damage and loss evaluation, there were communication problems, and there was a lack of assignment of responsibilities.

In the first drill, the information on the selected communities and farmers came to the organisers after the extension agents had already left for the field. The regional office tried to communicate the change in location to the extension agents, but this proved difficult owing to the lack of coverage of the mobile network in the area. In one case, it took several hours to notify them, providing, inadvertently, an opportunity to observe communication‐related challenges. In the second drill, the selected farmers were not informed beforehand.

Field visits in both cases started late and took longer than expected. The execution of the drill was affected by poor cellular telephone coverage, impacting on communication between the coordinators and the extension agents.

During both events, extension agents did not apply a standardised instrument and method to evaluate damage in the field, although all were advised to use the same official MAGA survey tool. It seemed like every extension agent had his own way of collecting information and coming up with a monetary estimate of loss and damage owing to drought—all extension agents involved in the drills were male, even though MAGA employs many female extension agents. The extension agents asked a wide range of questions about agricultural production, including about the losses that other community members had experienced and the general situation of the household. They recorded this information on the back of the form. After both exercises, they prepared a report that included some details of the calculations and a number of photographs.

Extension agents included an estimation of losses due to pests in the second drill, pointing out the presence of white grubs (larvae of *Phyllophaga spp.*). The total estimated included losses owing to low germination, white grubs, and the simulated drought.

Farmers’ influence on extension agents’ damage and loss evaluations was observed in the second drill. Farmers felt that they would receive more help if they reported more crop damage. In addition, MAGA representatives were seen to be correcting and influencing the results of the extension agents’ field observations of crop damage and losses after activities had concluded. Hierarchical rank and seniority in MAGA played an important part in influencing the results, with a senior person overruling the judgements of the extension agents.

The extension agents addressed the male head of the household to obtain information. This is logical given the information required: primarily on staple crop production losses. But this might paint an incomplete picture of the impacts of drought as women usually have their own domain within the farm household, such as a kitchen garden or poultry production. Although this does not change the official information on basic grains, it helps in developing a clearer opinion on how serious the drought is in relation to food security.

## Discussion

This was the first time that MAGA Chiquimula implemented the PIR for drought‐related emergencies. Consequently, the institutions and participants had no experience on which to build.

The drill produced four key findings. First, it pointed up a wide range of institutional issues and problems in implementing the PIR. The PIR is clear on hierarchical relations (see Figures 1 and 2), but the protocol lacks specifications on horizontal coordination and communication mechanisms and on inter‐institutional interaction at the local level between MAGA and other important drought response bodies. Furthermore, it lacks indications on how the information is transferred back to the local level once evaluated by central decision‐makers.

The drills revealed deficiencies in the training of MAGA staff in PIR procedures. There is little transparency and regulation of the process that leads to the regionallevel report. The PIR does not provide a standardised format for gathering information in the field. Although MAGA has an institutional form with which to collect damage and loss data, extension agents have not been trained in its use, and there is no homogeneous approach in place for damage evaluation; the result is influenced, therefore, by personal opinion. This leads to problems in the quality and reliability of the data and makes precise and target‐oriented decision‐making more difficult at all levels. The absence of standardisation, harmonisation of processes, and division of tasks are considered to be important factors hindering effective communication during a disaster response (Bharosa, Lee, and Janssen, 2010).

It is clear that the lack of standardisation in the use of survey instruments spawned visible problems in subsequent phases of the execution of the PIR. In one instance after the drought of 2014, a MAGA departmental office reported that more families were affected by drought than the total number of families living in that area according to the official census. This is an obvious error that probably reflects political interference in reporting.^9^ Even though these types of issues are corrected to the extent possible at the central level, this example makes it clear that issues concerning data collection and transmission, as well as external influences, have far‐reaching ramifications for the accuracy of data.

Second, the drill facilitated the meeting of different institutions (CONRED and MAGA, inter alia) at the local level and with other hierarchical levels of MAGA. This is important because drills in other contexts reveal that information‐sharing and coordination between agencies are crucial but complex and difficult tasks (Bharosa, Lee, and Janssen, 2010). Intra‐institutional collaboration at different scales has been recognised as an important tool in proactive drought management (see, for example, Econnics, Victoria, BC, 2016).

Third, the drill facilitated reflection and open discussion on the problems that became evident during the events. Such reflection is not possible by regular staff in a normal employment routine owing to time pressure and work overload. Reflection and the construction of organisational and individual knowledge are important components of organisational learning processes that aim to alter the behaviour of individuals and organisations (Manz and Sims, 1981).

Fourth, the drill led to concrete recommendations from the participants about improving the implementation of the PIR. These proposals flowed from a common experience and were applicable to individuals in a wide range of agencies and at different hierarchical levels. This finding confirms that debriefing the participants after the event allows drills to generate new, useful knowledge (Crookall and Thorngate, 2009).

The exercise also revealed several obstacles to successful implementation of an emergency drill for institutional disaster preparedness. The most important one was the lack of any mechanism for accumulative learning. Although participants rated the initiative as an excellent opportunity for reflection and capacity‐building, there was not much evidence of institutional learning processes or behavioural change between the first and the second drill. MAGA did not take up any of the lessons learned or recommendations. Follow‐up with key informants after the second drill suggested that it too did not trigger any sustainable learning processes or changes in the institution. MAGA has a high rate of staff turnover and a deficient information management infrastructure. These factors inhibit the successful use of emergency drills for institutional capacity‐building.

A proper debriefing straight after the event was not enough in this case to ensure that experiential learning translates into behavioural change in the institution. To a certain degree, the long‐term learning outcome of a drill or simulation is not predictable and depends on a variety of variables (Hofstede, de Caluwe, and Peters, 2010). The cultural and institutional environment generally plays a significant role in how drills influence long‐term learning in emergency response (cf. Pelling, 2007; Hofstede, de Caluwe, and Peters, 2010). In Guatemala, the institutional weakness of the public sector produces a lack of continuity in political programmes and the development of personnel. Moreover, one should note that the second drill in 2015 took place a few months before the presidential election in the country. Potential changes in the personnel structure, in programmes, and in budgets might be reflected, therefore, in the self‐assessment of the participants.

## Conclusion

The observations support the conclusion that drills are a useful and effective instrument with which to evaluate the organisational capacity of public institutions to respond to emergencies caused by agro‐climatic developments. Specifically, they can be used to assess the institutional response to slow‐onset events such as droughts.

The drills provided a secure setting in which to make problems tangible, to trigger reflections on institutional emergency response capabilities, and to motivate staff and stakeholders to discuss solutions to the problems encountered. While these are valuable contributions, the drills were not transformative: they did not yield changes in MAGA's institutional behaviour or drought response protocols. In part this is because of structural problems in the Guatemalan public sector related to the short political planning cycle, making substantial changes at the institutional level very difficult to implement.

Some of the challenges are specific to the setting, but the difficulty of transforming knowledge and experiential learning gained through the drills into long‐term behavioural changes at the individual and organisational level probably is a generic problem. Drills and simulations are valid tools for creating capacity and knowledge, but the institutional context majorly influences whether or not they can contribute to sustainable organisational learning that spawns behavioural change.

Embedding drills that focus on drought in a broader strategy aimed at creating and improving the institutional capacity of disaster response is a key recommendation of this study. This process should include developing improved protocols that pay more attention to inter‐sectoral linkages and training technical staff properly in the application of the new protocols. Changes are needed to make it possible to retain trained personnel for longer periods. As this will take time, in the short term institutions need instruments to train new personnel quickly and to ensure compliance with data collection standards. Digital media and instruments can play an important role in achieving better training. The need for stronger inter‐sectoral coordination implies that CONRED, the government institution responsible for inter‐sectoral coordination of disaster response, exercises leadership in fashioning disaster response protocols and in enhancing institutional capabilities.

Another key recommendation is that MAGA undertake a capacity‐building process among its extension agents, accompanied by a process to standardise steps for damage and loss evaluation and the gathering of information. CONRED and MAGA should view emergency drills as a valid tool with which to establish the institutional capacity required for an adequate response to slow‐onset disasters and for fostering institutional learning, and they should put in place mechanisms to fulfil this objective. As part of such a wider strategy, drills would make an important contribution to integrated drought risk management.

## Acknowledgements

This research was made possible by the financial support of the Inter‐American Institute for Global Change Research (grant number CRN3107) and was implemented as part of the CGIAR Research Program on Climate Change, Agriculture and Food Security (CCAFS), which is carried out with the support of the CGIAR Trust Fund and through bilateral funding agreements. For details please see https://ccafs.cgiar.org/donors (last accessed on 3 October 2018). The views expressed in this document are those of the authors and cannot be taken to reflect the official opinions of these organisations.

## Endnotes

1 In this paper, the term ‘drill’ is used to denote the type of simulation carried out in real time and involving physical aspects of a real‐world emergency response.

2 Also see the Global Hunger Index of the International Food Policy Research Institute at http://ghi.ifpri.org/ (last accessed on 17 September 2018).

3 Guatemalan Ministry of Agriculture, Livestock and Food in English.

4 National Institute for Seismology, Vulcanology, Meteorology and Hydrology in English.

5 Directorate of Geographic, Strategic and Risk Management in English.

6 Sub‐directorate of department dependencies in English.

7 ACF is an international non‐governmental organisation (NGO); CATIE is a Latin American tropical agricultural research and higher education centre; and Bioversity International is a global research‐for‐development organisation.

8 Thanks to an anonymous peer reviewer for pointing this out.

9 Personal comment made by a MAGA civil servant.
